# Intoxication in a Colombian mercury mine: Aranzazu, 1948-1975

**DOI:** 10.1590/S0104-59702024000100048en

**Published:** 2024-10-11

**Authors:** Juan-Sebastián Bonilla, Alvaro J. Idrovo, Helwar-Hernando Figueroa

**Affiliations:** i Master in History, Escuela de Historia/Universidad Industrial de Santander. Bucaramanga – Colombia bsebastian98@gmail.com; ii Titular Professor, Departamento de Salud Pública/Escuela de Medicina/Universidad Industrial de Santander. Bucaramanga – Colombia idrovoaj@yahoo.com.mx; iii Titular Professor, Escuela de Historia/Universidad Industrial de Santander. Bucaramanga – Colombia helwarff@uis.edu.co

**Keywords:** Colombia, Aranzazu, Mining, Mercury, Poisoning

## Abstract

This study analyzes the activities, poisoning, and medical treatment of the “La Esperanza” mercury mine workers in Aranzazu (Caldas, Colombia) between 1948 and 1975. The mining work there was difficult due to the geological instability of the area and the use of inappropriate mining technology, which generated a high occurrence of poisoning manifested in tremors of limbs, bleeding gums, loss of teeth, and bad mouth odor. The mine was the first Colombian company to treat its poisoned workers with dimercaprol and penicillamine. Occupational toxicology in Colombia was thus born during one of the most significant occupational health disasters in the country.

Mercury intoxication is an occupational disease especially frequent when mining activities are carried out with inadequate occupational health conditions (Parsons, Percival, 2005). Since ancient times, intoxication problems in mercury mines were amply noticeable due to their clear clinical manifestations. In this sense, in his famous work *De morbis artificum*, Bernardino Ramazzini (1633-1714) noted that “It is from the mercury mines that the cruelest curse of all those that treat and destroy miners comes from”^1^ (Ramazzini, 1964, p.21). Then Ramazzini quoted Gabriele Falloppio’s (1523-1568) treaty *Metals and Other Minerals*, where the author reported that mercury miners lasted barely three years, and Michaelis Ettmüller’s (1644-1683) book *Mineralogia*, where he described how within four months intoxicated miners presented with limb or general paralysis and vertigo caused by “mercurial fumes” (Ramazzini, 1964, p.21). In remote times, the name given to this kind of intoxication was hydrargyrism, from the Greek term *hydrargyros* (ὑδράργυρος) for mercury, which we will use here from now on, as this is how it was known in the Aranzazu mine in Colombia.

The disease is characterized by erethism, tremors, and stomatitis, and reports show that it is frequent in mercury mines worldwide. It has been documented in old mines in Almadén (Spain), Huancavelica (Perú), Idrija (Slovenia), and others (Nriagu, 1994), where mercury is found in the form of cinnabar (HgS, a combination of mercury and sulfur). In the case of native mercury (the name of the metal when it is found in the liquid form, with no presence of other elements), exposure is much higher because it is easier for the metalloid to evaporate inside the shafts and generate high-exposure environments (Kobal, Dizdareviĉ, 1997). In all these contexts, erethism usually appears first with severe behavioral or personality disorders; tremors are more frequent in the upper limbs when directed and purposeful motor movement is involved (intention tremor), and stomatitis manifests itself with excessive salivation and gum sensitivity (Buckell et al., 1946).

Here we argue that the exploitation of the Aranzazu native mercury mine (Caldas, Colombia) was permeated by the high occurrence of hydrargyrism over a few years. This happened shortly after the issuance of the Substantive Labor Code (Código Sustantivo del Trabajo) in 1950 (Decree n.2.663), where mine administrators were requested to regulate miners hiring and offer them optimal work safety conditions (Colombia, 1950). Before the closing of the mine, the disease was treated empirically using chelating medications (metal and metalloid antagonists that favor excretion from the body) and following the dosage guidelines defined by the company’s official physician. Although hydrargyrism prevention, diagnosis, and treatment were based on the scientific literature on several mercury mines around the world, this was mainly an experimental therapy as at the time the use of chelating agents was not standardized.

## Historiographic studies of occupational disease

At the beginning of the twenty-first century, Diego Armus (2002) pointed out that the study of disease was gaining a prominent ground in Latin American historiography due to the fragmentation of the main historiographic trends and the attention that social and human sciences have bestowed on disease as a research subject in the history of medicine and public health. The author argues that the history of disease has developed based on three topics: the social and political dimension of epidemics; the external influences in medical and scientific development and regional public health policies, and the cultural dimension of disease. A series of studies have focused on workers’ diseases, precisely our research field.

Pioneers such as George Rosen (1985) and Paul Weindling (1985) have contributed significantly to the history of work diseases and occupational health by setting its fundamentals through studies conducted in various countries. In Spain, for example, Cohen and Ferrer (1992) analyzed ancylostomiasis and silicosis in mining zones, and Menéndez Navarro (1992) explored the association of work, health, disease, and care offered to miners intoxicated at the Almadén mercury mine. Doubtlessly, the studies conducted in this mine are essential to understanding the Aranzazu events, notwithstanding the marked differences arising from the differing chemical forms of mercury in the two sites. This contrasts with other studies in Latin America that have paid more attention to institutional issues and hegemonic discourses concerning medical activity both in Brazil (Lacaz, 2007; Almeida, 2008) and Argentina (Grimberg, 2000).

Colombian researchers have documented the history of diseases affecting certain workers. Health issues, however, have not been a priority in workers’ movement fights, and protests have centered around poor medical care and compensation mechanisms when injuries or occupational diseases occur (Torres, 2022, p.67). Some of these studies focused on oil industry workers (Quevedo, 1996; Quevedo et al., 2004; Luna-García, 2010), coffee industry laborers (García, Quevedo, 1998; García, 2007; Urrego Mendoza, 2016), railway employees (Restrepo, 2004), and miners. Among the latter, of interest in the present work, uncinariasis and tuberculosis have been analyzed in gold mines in Antioquia (Gallo Vélez, 2010; Gallo Vélez, Márquez Valderrama, 2011a, 2011b; Ossa Viana, 2019), and most of them have revolved around infections defined as “tropical diseases” in agreement with publications on the history of Latin American public health (Cueto, Palmer, 2015).

Given their relevance to our study, the history of respiratory illnesses among miners is of special interest, as these lead directly to occupational diseases associated with agents occurring in the working environment and, therefore, the subject of occupational medicine (Kennedy, 1994). Oscar Gallo Vélez (2010) has developed this line of research in his studies on mine workers, where he emphasizes the difficulties of silicosis diagnosis and its relation to work. His studies focus on the “El Zancudo” gold mine in Titiribí, Antioquia, where respiratory diseases abounded. The historical importance of this mine relates to its economic relevance for more than a century. According to Titiribí’s hospital records and death certificates between 1865 and 1948, respiratory diseases were frequent, and physicians tended to associate them with tuberculosis. Only in the 1950s, they started to diagnose silicosis among miners (Gallo Vélez, Márquez Valderrama, 2011a, 2011b). This bias in medical diagnosis should be understood in the context of epidemiological realities during the first half of the twentieth century in the country when tuberculosis incidence increased rapidly (Idrovo, 2001). Similar disputes occurred in other parts of the world where, intentionally or not, pulmonary tuberculosis concealed silicosis, mainly due to the disease’s chronic evolution (McIvor, Johnston, 2016).

The history of occupational diseases and occupational medicine is the framework of the study on the intoxication in the Aranzazu native mercury mine. Historical reconstruction has evidenced the high occurrence of acute hydrargyrism in this mine, not described before in the country. Although the exposure to mercury in the air in gold extraction sites in Colombia is deemed as the highest *per capita* worldwide (Cordy et al., 2011), the incidence of hydrargyrism is quite low (Zapata Díaz, Mesa Arango, Berrouet Mejía, 2020). Recent studies suggest that the antagonism generated by geological selenium (Varona-Uribe et al., 2023), abundant in some regions of the country (Ancizar-Sordo, 1947) and also traceable in certain foods (Córdoba-Tovar et al., 2022; Díaz et al., 2023), offers protection against metals or metalloids intoxication. Cases associated with organic mercury exposure from fish ingestion (De Miguel et al., 2014) are also scarce, although many nonspecific symptoms and signs are evident among the population exposed.

We intersected various primary sources from Colombian archives and libraries in our study. We accessed the mining licenses authorized by the Ministry of Mines and Petroleum (1953-1970) – presently Ministry of Mines and Energy – kept in the General Archive of the Nation (Archivo General de la Nación) and the correspondence of the Presidency of the Republic of Colombia with Mines ministers. In the Luis Ángel Arango Library and the National Library, we consulted *La Patria*, the newspaper with the largest circulation in the department of Caldas where the mercury mine was located, and the newsletters published by the Ministry of Mines and Petroleum. In the National Geological Survey library, we found maps and technical reports related to the “La Esperanza” mine, and we had access to administrative documents in the municipality of Aranzazu town hall and city council. However, the most productive information source was the 15 interviews with former miners and their families that we later contrasted with the documents we retrieved from the archives mentioned before. We also had access to audiovisual material from the independent cultural collective “Aranzazu up to date” (Aranzazu al día) and to the interviews they conducted during the last ten years. Then, the information was systematized using the free access software Zotero to collect, cite, and synchronize hundreds of data sets and historiographic discussions. Our study adhered to the guidelines set for research with humans in Colombian regulations and the Helsinki Declaration and was approved by the Universidad Industrial de Santander ethics committee for scientific research on June 7, 2019.

## Aranzazu and the “La Esperanza” mine

Several Colombian regions have cinnabar deposits with mercury in low proportions, among them Aguadas, El Limón (La Merced), and Samaria (Salamina) in the department of Caldas (Brooks, 2012) used for gold extraction since pre-Columbian times (Brooks, Schwörbel, Castillo, 2011; Brooks, Bermúdez Restrepo, Cadena, 2016). According to the mercury measurement results in hair from pre-Hispanic samples (Idrovo et al., 2002) and the historical analysis of the metallurgy developed at the time (Idrovo, 2005), mercury exposure was minimal. The “yard process or method” implemented by Spain in its colonial territories during the sixteenth century was used for mercury amalgamation with gold and silver (Nriagu, 1993, 1994). During colonial times, the mercury used in Nueva Granada (Colombia today) came from the Almadén (Spain) (Higueras et al., 2011) or the Huancavelica (Perú) mines (Brown, 2001; Cooke et al., 2009). All mining activities in Colombia requiring the use of mercury depended since then on imports from other countries; this changed temporarily during the period the Aranzazu mine was active.

**Figure 1:**
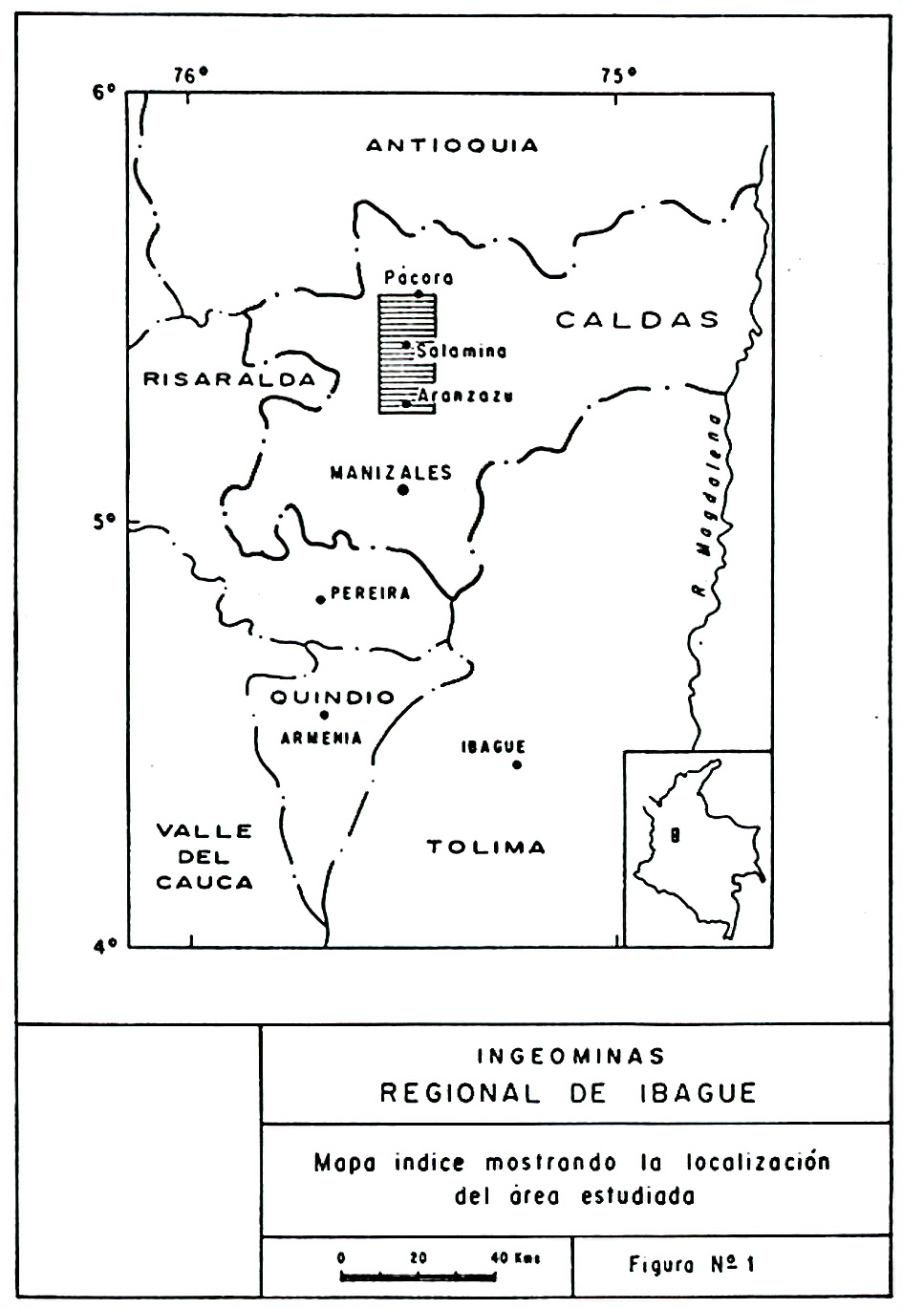
: Map showing the location of the “La Esperanza” mercury mine (Lozano, Pérez, Vesga, 1984)

Aranzazu municipality was founded in 1853; it borders the municipalities of Salamina, Filadelfia, Marulanda, and Neira and is located around 50,5km from Manizales, the capital of the department (Figura 1). In the decades of 1910 and 1930, during the coffee growing boom, it had a privileged position, especially since the construction of the cableway that connected Aranzazu and Manizales and operated from 1928 to 1942. In 1922, street lighting was installed (Valencia Llano, 1996), denoting the importance that the municipality’s location had for coffee transport in the area of greatest national production at the time.

The “La Esperanza” mercury mine was located in a wooded area of the Andes Central Cordillera, in El Manzanillo village, jurisdiction of Aranzazu, department of Caldas, Colombia. The territory has mountains, valleys, and plateaus with varying altitudes from 1400 to 3500masl. As for its geology, it is a transition zone between the continental and oceanic crusts presenting intense folding, faulting, uplifts, and batholithic intrusions of great magnitude that confer a marked structural and stratigraphic complexity to the area (González Iregui, 2001). The anomaly favoring the presence of mercury is related to the Romeral fault system: the “La Esperanza” mine is located in the southernmost region of this geological belt (Lozano, Pérez, Vesga, 1984). The mine extension was almost one thousand hectares bathed by the La Honda, Dantas, and El Sargento streams on a terrain prone to constant landslides, which hindered the mineral extraction process (Licencia 1540, 17 mar. 1959).

The history of the mine may be divided into two stages according to the way the metalloid was extracted, which had direct consequences on the exposure levels of mercury in the air and, therefore, on their adverse effect on workers’ health. During the first stage, between 1948 and 1961, open pit mining with the use of basic tools prevailed. The mining companies in charge of the extraction during these years were: Roberto Botero, Consorcio Minero and Walter Ringueski (1948-1956), Borrero and Robledo (1957), and Chocó Pacífico (1958-1960). The second stage, from 1962 on, was characterized by underground work and the participation of the Colombian Miners Consortium, a lawyers union specialized in mining law that obtained licenses from the Ministry of Mines and Energy and then sublet the mine’s administration. This resulted in various companies leading the mercury extraction operation at that time: Merco (Mercurio Colombiano) from 1963 to 1965, Compañía Nueva Esperanza and Southern Union from 1965 to 1970, Américo Marán from 1970 to 1971, and Compañía Mercurífera de Caldas from 1972 to 1975 (Lozano, Pérez, Vesga, 1984).

In 1948, in the lands of Mr. Roberto Botero, peasants found native mercury amidst quartz, calcite, and pyrite stones with slight impregnations of cinnabar (Lozano, Pérez, Vesga, 1984). It is worth noting that this type of mercury is different from that found in cinnabar mines, such as those of Almadén, Huancavelica, and most of Idrija. This mercury, infrequent in world geology, implied a less technical and quicker extraction, as well as less economic investment. The uniqueness of the Aranzazu mine can only be compared with the Quicksilver Creek native mercury mine in Central Victoria, Australia (McQueen, 2011), and the Clear Creek mines in San Benito County, California, United States of America (Dunning et al., 2005), both closed now due to their high toxic potential and geological threat (Abbas et al., 2015).

From 1948 to 1960, the miners were the peasants from Aranzazu and the neighboring towns who labored by shifts during the day with a pick and shovel and in the open, looking for the mineral in big holes, under the stones, and even the trees. This work on the surface had no direct repercussions in the form of intoxications despite the informality of the work: no use of adequate helmets, gloves, or boots, which involved the breach of the occupational health requirements stated in the Substantive Labor Code. Notwithstanding, it was during this period that a camp equipped with dining rooms and cots for the miners’ rest was built with guadua and zinc (Orozco, mar. 2023).

**Figure 2:**
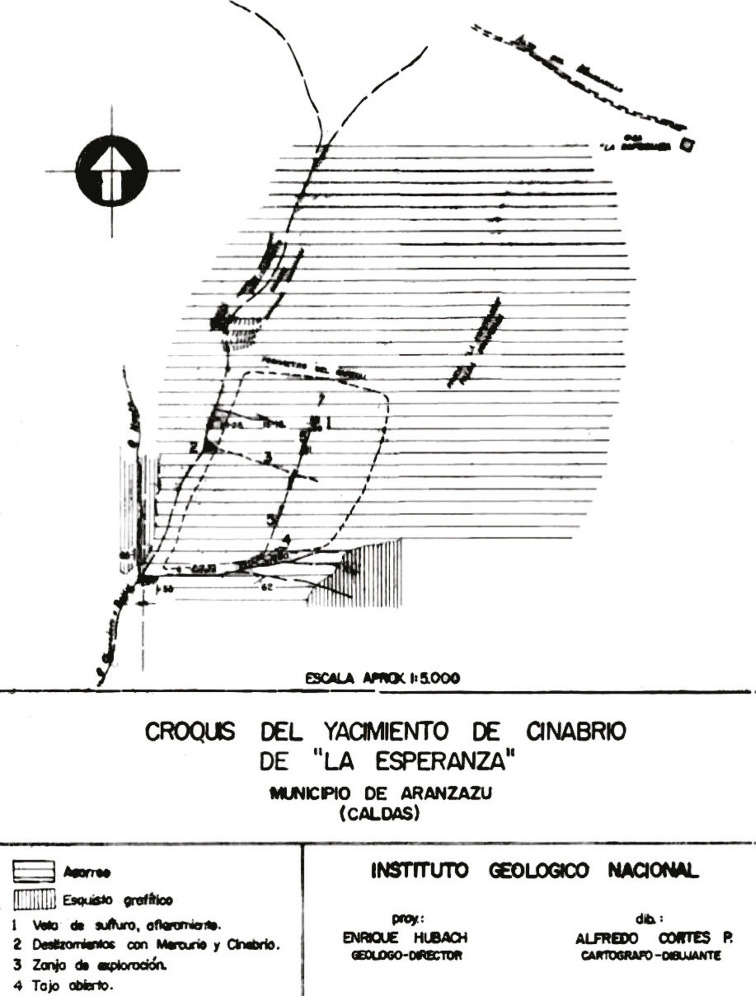
: Sketch of the “La Esperanza” cinnabar deposit (Hubach, 1951)

At the end of the 1950s, the activity on the surface changed with the construction of the first four shafts (Figure 2); however, an engineering technical report from 1959 pointed out that:

No maps of the excavations have been made showing their locations, cuts, directions, and slopes, which makes uncertain the positive results of these works. Supporting these tunnels is difficult given the quality of their pillars and the porosity of their soil; the almost total shortage of wood worsens the problem (Informe…, 1959, p.4).

The first tunnel to be built generated the most expectations for mine administrators, as it promised excellent performance due to the high quality of the mercury and the mineralized cinnabar shales found, although soil porosity implied constant landslides. The second, third, and fourth shafts were built in response to temporary situations in the “fruitless hope to find again the vein detected in tunnel n.1,” but the results were not satisfactory enough due to the difficulties of the terrain (Informe…, 1959, p.3).

Already in the second stage, between 1964 and 1967, the fifth and sixth tunnels were built with an average area of “one hundred or 150 meters” (Naranjo, mar. 2023). When the construction finished, all shafts had light, rails for moving rocks, and electric ventilation through plastic tubes (Lozano, Pérez, Vesga, 1984). In this stage, the underground work was already more specialized, and the payment miners received depended on the activities they carried out. Those in charge of surface activities received sixty pesos a week while those working in shafts received between four to five hundred pesos per week, which was far above the salary for agricultural activities, of seven to eight pesos per day (Orozco, mar. 2023). Briefly put, working in the “La Esperanza” mine meant a wage increase for these miners that augmented exponentially when their activity was underground.

**Figure 3:**
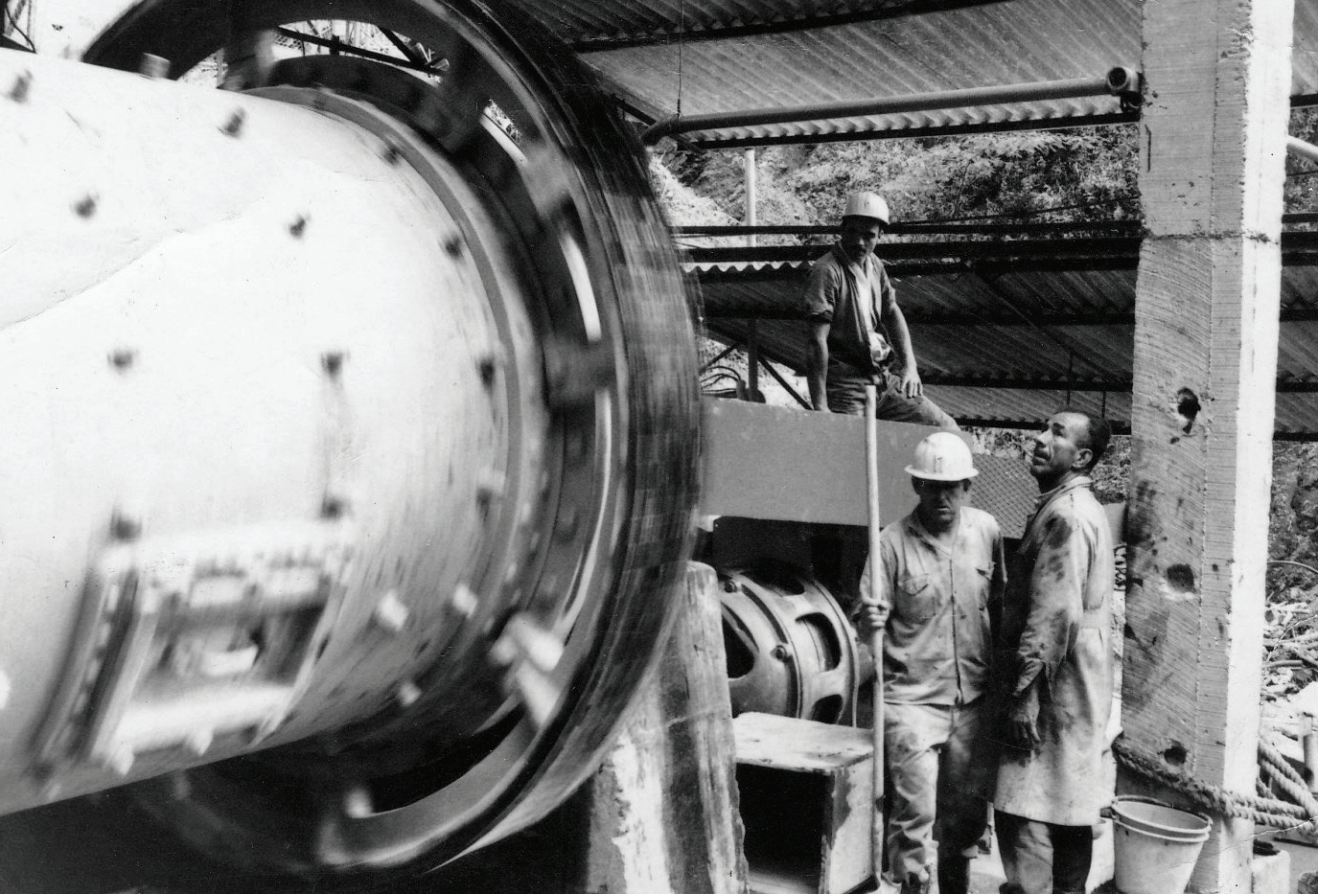
: Miners from the “La Esperanza” company working on the surface (Duque, s.f.a.)

**Figure 4:**
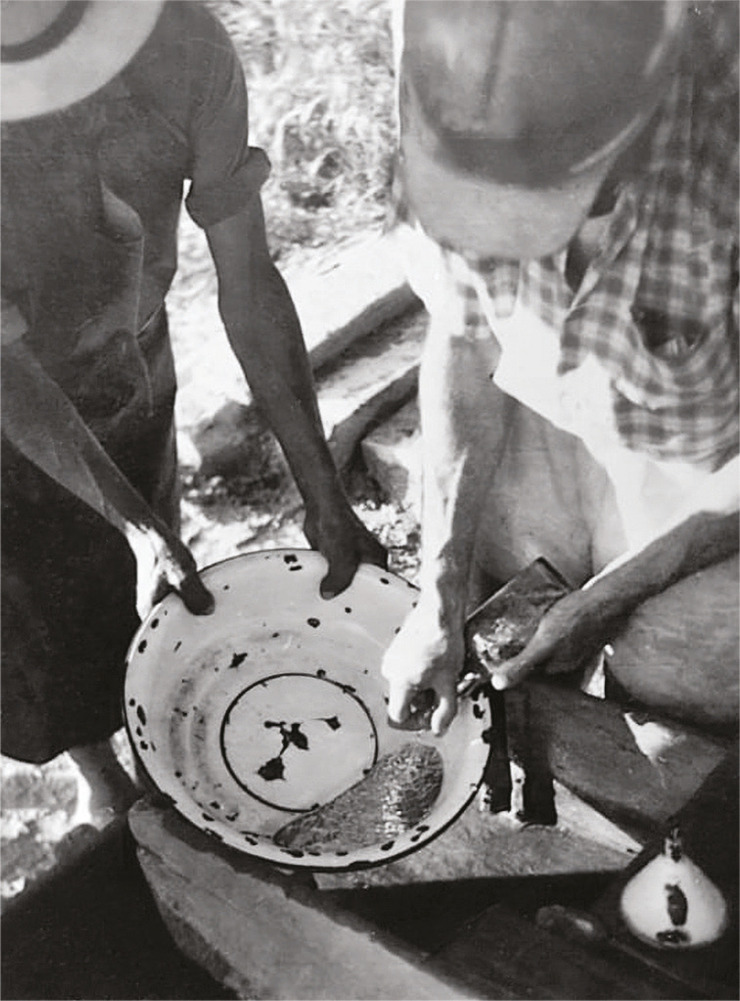
: Miners from the “La Esperanza” company beating the mercury (Duque, s.f.b.)

The work in the shafts was done in groups of four miners in shifts of six to eight hours; two o the miners worked chipping rock material with small picks while the other two drilled the tunnel with dynamite; the dangers of such activity became evident in 1966 when an explosion left two miners wounded (Heridos…, 4 jul. 1966). The rock material was removed from the tunnel by the cart operators who transported it to the washing hopper, the biggest equipment in the mine, where the rocks were cleaned with pressurized water to separate them from useless materials such as sand and mud (Figure 3). Finally, the rock was taken to a jaw crusher where it was ground up; mercury was then taken to a small hopper provided with channels to be packed in steel bottles (Figure 4) (Orozco, mar. 2023; Gallego, mar. 2023).

The amount of mercury extracted in the second half of the 1960s was considerable. In 1969, for example, the mine produced 23,734 pounds of mercury that were sent to Bogotá and Medellín (López Agudelo, 1971, p.18). However, miners’ working conditions were unsuitable, as the site was “a swamp and it smelled bad, it was pure mud” (Soto, mayo 2023). Miners at the Aranzazu mine did not enjoy basic occupational health conditions to reduce intoxication levels; besides, “the camp is located in an inappropriate place, below the site of present mining activities and, therefore, workers are subject to intense contamination outside working hours and, naturally, during their workday contamination is even greater” (Morer, Nicholls, 1960, p.22).

Although there is no data regarding mercury exposure inside the shafts, the evidence from similar mines is useful. In the mine in Idrija, Slovenia, for example, around 70% of the mercury was found in the form of cinnabar and the other 30% as native mercury. In not-ventilated native mercury mines, mercury concentrations were extremely high, even over 3mg/m^3^ in the air, associated with many hydrargyrism cases (Kobal, Dizdareviĉ, 1997). A similar situation was recorded in the Almadén mine located in Spain. This mine contains mostly cinnabar, and while drilling through the rock, the concentration of mercury in the air used to reach 2.26mg/m^3^ (García Gómez et al., 2007). The short time passed until the first cases of hydrargyrism appeared in Aranzazu evidences a very high exposure. In this sense, Alfredo Menéndez Navarro (1996, p.151) describes how in the Almadén mine, the tremors and the stomatitis appeared as a “chronic type of process,” contrasting with the few weeks miners working at Aranzazu needed before presenting with mercurial intoxication signs.

However, the extraction process itself resulted in a constant loss of mineral. Geologists visiting the mine in the 1950s and 1960s pointed out that the equipment used was intended for gold extraction and, therefore, “the main defect of this small mining site is that after crushing the mineral, it is still too coarse to liberate all the native mercury it contains. It is difficult to know exactly how much mercury is lost in the mill dumps, but it may amount to a 20% to 30% loss of mercury” (Morer, Nicholls, 1 dec. 1958, p.9). Other authors indicate that the loss percentage reached 50%, meaning that “the wealth dilapidated is approximately the same as the benefits obtained” (Informe…, 1959, p.8). In fact, this loss of mercury generated work for the “barequeros” dedicated to washing the sand in pans in El Chupadero and El Roblal streams adjacent to the mine. The “barequeros” were subsistence miners who profited from the mining waste by collecting mercury in rudimentary ways and then bottled it in glass containers (Alegría, mar. 2023). Despite their precarious working conditions, probably their mercury exposure was not very high, as they worked in the open air and this protected them against high concentrations. Also, the “barequeros” were a more diverse group of workers, including minors and women, while miners were exclusively adult men (Martínez, mayo 2023). Although the profit for “barequeros” was much less than for miners, they collected enough mercury to improve their families’ life conditions.

## Acute hydrargyrism: occurrence and clinical presentation

Hydrargyrism symptoms in Aranzazu depended on mercury exposure levels. The highest exposure was that of the miners working inside the shafts. After a few days, they showed the first clinical manifestations; then were those working on the surface and, lastly, the “barequeros,” with the lowest exposure. According to the miners’ testimonies, the first symptoms were the loss of appetite, headaches and bone aches, fatigue, and paresthesia, all compatible with mercurial erethism. In many cases, these symptoms were so evident that the ladies in charge of the kitchen were able to detect the sick just at sight (Salazar, mayo 2020) and they were the ones who alerted the administrators about the physical condition of the miners. When the exposure increased, so did the physical and mental symptoms of the sick. The miners usually had nausea, diarrhea, bad breath, and gum pain, and their teeth became brownish. Jesús María Álzate (mar. 2023) recalled how “my teeth were loose. Just imagine, my teeth started to damage and already at 16, I had to use a denture.”

In a matter of days, the most evident sign of hydrargyrism appeared: the intention tremor, which ensued in waves, with interruptions that lasted some minutes and came back strongly and suddenly. The movement started in fingers, lips, and eyelids and then extended progressively to the limbs (Gutiérrez, 1997). These tremors hindered workers’ daily activities. In this sense, Damaris, the daughter of a miner, remembered that her father needed help for daily life basic activities such as using cutlery, taking his breakfast, and bathing (Soto, mayo 2023). Reinaldo also recalled that there were many sick miners:

People were seized by tremors and couldn’t even stand. They couldn’t even stand still. Once they grabbed you, you couldn’t even eat. You took the spoon and tried to take it to your mouth and when finally you managed, it had nothing. Nothing was there in the spoon. The same happened with coffee. I remember that when I started with the tremors, I used to return to Aranzazu to have a coffee, but if I had no one to help me, I could not drink it (Orozco, mayo 2023).

Another symptom associated with hydrargyrism was the neuropsychiatric manifestations. Historically they have been known as “mercurial erethism” (Parsons, Percival, 2005), expressed as rapid changes in individuals’ personalities and psychic disorders like depression, unmotivated crying, memory loss, manic-depressive psychoses, and irritability (Gutiérrez, 1997). Mrs. Marina Gómez (abr. 2023) recalled how her husband, incapable of sleeping, experienced constant emotional susceptibility, fluctuating between extreme sadness and joy, together with depressive periods. However, we must point out that maybe erethism due to mercury intoxication did not stand out so much in Aranzazu, as the population there has one of the highest incidences of bipolar affective disorder in Colombia associated with endogamy among its inhabitants since the end of the nineteenth century (Bedoya et al., 2006; Jordán-Quintero et al., 2022). Probably, the most striking manifestation was the tremors that the respondents related unmistakably to the work in the mercury mine.

Anyway, this combination of symptoms due to mercury intoxication seriously deteriorated several people’s health, first, because in the first years of the mine operation, the medical treatment against hydrargyrism was not known and, second, the miners used to consume considerable amounts of alcoholic drinks despite their weak physical condition. In this sense, Rubiel Álzate (mar. 2023) pointed out that “people dried up, they were left in bones and took to drinking in the town bars.” Renet Giraldo (mar. 2023), son of the owner of the tavern closest to the mine, recalled how miners “came here [the tavern] to have a beer, and their glasses had to be half-filled because otherwise, their hands started to tremble.”

To calculate the number of miners affected by hydrargyrism we used the testimonies of mine exworkers and the physician Hernando Marín Maya (1941-2021), hired in 1967 by the Consorcio Minero company administrative board to treat the disease. Marín claimed that when he started to work at the mine, around 95% of the miners had hydrargyrism and his interventions managed to reduce this figure sensibly. In his own words, “95% were affected by mercury intoxication” (Maya, mayo 2020); there is no doubt that his presence marked a before and after in terms of the number of hydrargyrism cases. In the first years of the mine operation, probably there were no significant changes in the occurrence of intoxication cases and, possibly, it was the same observed when underground activities were still taking place. In contrast, at the time before the mining activities ended in 1975, surely hydrargyrism cases dropped significantly. Another mine administrative ex-worker remembers that “if there were one hundred men, I would say 25 were sick” (García, mar. 2023).

An approximation to the workers’ risk of being diagnosed with hydrargyrism demands determining the total number of miners; this can be estimated based on the direct testimonies of several of those who worked there during the last ten years of the mining operation. One of them reported that “at the time I worked there, we were 250 workers” (Orozco, mar. 2023); this was confirmed by other miners who remember they were “around one hundred workers, more or less” (Naranjo, mar. 2023), and that they were “many, more or less one hundred, because we worked in shifts” (Soto, mayo 2023). The last of the mine investors also agrees with the number of workers: “I had up to 120 there” (Marán, jul. 2018).

One of the reasons for the discrepancies in the number of workers was the variety of activities that took place in the mine, because “we were many workers, about one hundred or so, because there were three shifts, some leveraging, others loading the carts to transport the material, others grinding, and those were a lot because they had the grind all the material” (Agudelo, mar. 2023). Besides, it was clear from the interviews with former miners that their number was not constant: “There were many, indeed. Some fifty, one hundred, more or less. They already used machinery” (Álzate, mar. 2023). This suggests that the introduction of technology increased production and maybe also decreased the number of workers dedicated to activities outside the shafts. Near the end of the mine operation, the number of workers decreased: “Including Don Américo, there were about thirty, few already. But before there were eighty to one hundred workers” (Agudelo, mar. 2023). However, the number of miners in underground activities may have been more constant given the impossibility of having many working at the same time in the shafts. “We were 25, this was about forty years ago, in the seventies if I am not wrong. We were 25 in all the shafts” (Gallego, mar. 2023).

These testimonies show the risk workers had of being diagnosed with hydrargyrism. The total number of workers in the “La Esperanza” mine varied between one hundred and 250 and almost a quarter of them (25 to sixty miners) were hydrargyrism cases. We should also remember the high worker rotation, as intoxication symptoms and signs appeared barely one or two weeks after the miners were hired, and the fear of acquiring the disease of those working in the shafts. This suggests that the risk of being diagnosed with hydrargyrism was almost 100% among the underground miners and around 25% taking into consideration all the mining activities. The cases should be multiplied by the years when no occupational health measures were adopted in the mine, which may be more than a decade. Based on these considerations and the testimonies of the miners and their families, we can say that hydrargyrism in the Aranzazu mine was one of the massive intoxication events with the highest incidence in Colombia, and there is a need for further studies to understand its impact on workers’ health.

## Medical presence and patients’ care

Although Colombian regulations included norms for mining activities as early as 1915, medical care for workers at the mercury mine included occupational health measures only during the last years of the mining operation, when law 57 of 1959 was adopted requiring compensation for work accidents that were later extended in the Substantive Labor Code to cover disease prevention. Only in 1966 medical care services were hired; until then, sick miners were informally treated at Aranzazu’s drugstore, which explains why no precise information was found regarding the type of medical care offered. This situation changed when in 1967 doctor Marín Maya was brought by the Southern Union Company to organize medical care services and implement occupational health measures at the mine. Among other actions, Marín Maya collected studies with the medical and technical reports from the Almadén and Huancavelica old mines and concluded that the best way to fight hydrargyrism was by preventing the disease and not only treating it. This line of argument was heatedly debated in the 1950s in Spanish scientific circles after the medical failures reported in the treatment of sick miners (Manzanares, Montes, 2011).

Influenced by the medical advances in the Almadén mine, Marín Maya proposed an occupational health strategy to reduce intoxication levels among the Aranzazu miners. Initially, the miners had to comply with mandatory hygiene norms: smoking, alcohol drinking, or arriving to work with a hangover (veisalgia) were forbidden, and the uniforms and work implements should be cleaned. These norms were posted in wooden billboards in visible places and the workers who infringed them were fired (Maya, mayo 2020). Another measure included medical examinations three times a week, on Mondays, Wednesdays, and Fridays (Maya, mayo 2020). Miners were examined to detect anomalies indicating hydrargyrism; the information was then transferred to their medical records, and the sick received support for their recovery. The treatment included protein diets (high beef and milk consumption) and cleaning of the body injured areas; besides, depending on the disease severity, work in the shafts was restricted and miners were sent home for as long as needed (Orozco, mar. 2023).

However, the medical care for sick miners did not cover all personnel. Damaris Soto recalled that her father was fired when he got sick, and his relatives had to take care of him:

At that time, we didn’t have cotton swabs, so we used a toothpick covered with cotton to clean tooth by tooth because mercury coated them with something like slime, and they were all loose, the gums were kind of rotten, and he had bad breath, that’s why every single tooth had to be cleaned and treated with the medication (Soto, mayo 2023).

Sometimes, sick miners did not receive any economic aid while incapacitated and, therefore, they had to look for another job or even migrate to other cities (Gómez, abr. 2023). As we mentioned before, this kind of situation resulted in the constant turnover of miners, which would mean a high incidence of hydrargyrism albeit the lack of support documents.

For the treatment of miners with hydrargyrism, an infirmary with six stretchers was set in the camp and a nurse was provided to care for acute patients and those who had no family in the region. This medical attention was offered until the miners recovered enough to be transferred to their homes or their cots in the mining camp (Orozco, mar. 2023). Usually, sick miners were given dimercaprol (British anti-Lewisite, BAL) and penicillamine, chelating agents that had been recently introduced in toxicological therapeutics. BAL was developed by the British army during the Second World War to counter the effects of Lewisite, a Nazi arsenic-based chemical weapon (Waters, Stock, 1945). The medication facilitated the excretion of mercury through urine; its adverse effects, however, were multiple: hypertension, tachycardia, nausea, vomiting, burning sensation in the mouth, hand paresthesia, feeling of tightness or chest pain, tearing, excessive salivation, rhinorrhea, sweating and abdominal pain (Gerhardsson, Kazantzis, 2015). It is worth noting that the first studies on the use of BAL in humans were conducted on individuals with syphilis, especially on African-origin patients (Modell, Gold, Cattell, 1946). The Aranzazu miners and their families were aware of this new treatment: “My father was able to receive the treatment, one day they arrived in the mine, he was conscious that it was an experimental situation, and he was among the first, not to say the first, who underwent the treatment they offered… and thank God he ended his days very well” (Soto, mayo 2023).

Penicillamine, a chelating agent resulting from penicillin hydrolytic degradation, was proposed by Walshe (1956) to treat mercury intoxication; it had fewer adverse effects than BAL (Parameshvara, 1967), in fact, only some individuals showed acute reactions such as fever, rash, blood dyscrasias, and, in severe cases, kidney damage (Gerhardsson, Kazantzis, 2015). Although the route of administration and dosage of the chelating agents used in the mine are not clear, articles published at the time suggested that BAL was more effective by the parenteral route while penicillamine could be administered by the intramuscular route (Pagnotto, Brugsch, Elkins, 1960). An ex-miner by the name of Reinaldo recalled that in the infirmary they used to inject him with “four daily shots, two in the morning and two in the afternoon, one of them in the vein and the other was intramuscular” (Orozco, mar. 2023), which suggest that both chelating agents were used at the same time. Specialized articles at the time emphasized that the medication should be administered three times in nine-day cycles, with one-week intervals between cycles (Parameshvara, 1967). In the end, Reinaldo recovered from hydrargyrism after three months of treatment, quicker than usual.

Sometimes, sick miners’ intoxication symptoms worsened, and they had to be transferred to hospitals in Manizales or Medellín. Although hydrargyrism does not usually result in death, it is possible that in some cases it worsened preexisting conditions; this explains why sick miners were forbidden access to the shafts, engaging them instead in open-air activities and insisting on the disinfection of uniforms, picks, and shovels. In any case, hygiene and daily life care practices such as no smoking and reducing alcohol consumption were encouraged (Marán, mar. 2023).

## Final considerations

In 1948, native mercury was found in Aranzazu (Caldas, Colombia) and the peasants in the region started to exploit it in the open air. When the first shafts were built and the Colombian Miners Consortium arrived in 1962, the mining operation concentrated on underground exploitations and a more technical extraction of the mineral. As there is no evidence of the use of respiratory protection elements, mercury exposure was surely very high inside the shafts, where the metal concentrated in the air. This meant that miners working in underground activities presented with hydrargyrism barely a few weeks after they were hired. Surface activities involved less exposure, but the risk of acquiring the disease was latent due to the lack of appropriate machinery and the adoption of gold-mining techniques that implied significant metal losses. Given the number of workers exposed to high mercury concentrations, the “La Esperanza” mine may well be the site of one of the main occupational health disasters in Colombia.

The arrival of the young physician Marín Maya in 1966 improved the situation. Hydrargyrism prevention and the adoption of mandatory hygiene and occupational health measures based on the experience at the Almadén and Huancavelica mines were the core interventions. This constitutes an example of the measures that should be implemented in these cases. In the “La Esperanza” mine, hygiene norms were posted forbidding smoking and alcohol consumption and encouraging the disinfection of the equipment and the uniforms. Besides, medical care services were established three days a week, and the sick were prompted to take a meat-rich diet and were treated with chelating agents (BAL and penicillamine) to increase mercury excretion through urine. These experimental medications in the 1950-1960 decade were used for the first time in Colombia to treat the Aranzazu miners affected by hydrargyrism with positive results. Former miners like Reinaldo Orozco recovered after three months of the intoxication. However, not all the sick had access to treatment, as many had left the mine and engaged in other economic activities.

## NOTE

1 In this and other citations of texts from Spanish, a free translation has been provided.
