# Supplementary material for: Intoxication in a Colombian mercury mine: Aranzazu, 1948-1975
Source: Hist Cienc Saude Manguinhos. 2024 Oct 11;31:e2024048. doi: 10.1590/S0104-59702024000100048en (PMC11472786; doi:10.1590/S0104-59702024000100048en)
Supplement: Translation

# Translation: Intoxicación en una mina de mercurio de Colombia: Aranzazu, 1948-1975

**DOI:** 10.1590/S0104-59702024000100048

**Authors:** Juan-Sebastián Bonilla, Alvaro J. Idrovo, Helwar-Hernando Figueroa

**Affiliations:** i Magister en Historia, Escuela de Historia/Universidad Industrial de Santander. Bucaramanga – Colombia bsebastian98@gmail.com; ii Profesor titular, Departamento de Salud Pública/Escuela de Medicina/Universidad Industrial de Santander. Bucaramanga – Colombia idrovoaj@yahoo.com.mx; iii Profesor titular, Escuela de Historia/Universidad Industrial de Santander. Bucaramanga – Colombia helwarff@uis.edu.co

**Keywords:** Colombia, Aranzazu, Minería, Mercurio, Intoxicación

## Abstract

Este estudio analiza el trabajo, la intoxicación y el tratamiento médico que recibieron los trabajadores de la mina de mercurio “La Esperanza” entre 1948 y 1975 en Aranzazu (Caldas, Colombia). La labor minera tuvo dificultades por la inestabilidad geológica de la zona y el uso de la tecnología minera inapropiada, lo que generó una alta ocurrencia de intoxicaciones, manifestado en temblores de miembros, sangrado de encías, pérdida de piezas dentarias y mal olor bucal. La mina fue la primera empresa colombiana que trató sus trabajadores intoxicados con dimercaprol y penicilamina. Así nació la toxicología ocupacional en Colombia, en uno de los más graves desastres de salud ocupacional en el país.

La intoxicación con mercurio es una enfermedad de origen ocupacional, siendo más frecuente en actividades mineras sin adecuadas condiciones de salud ocupacional (Parsons, Percival, 2005). Desde la antigüedad, el problema de la intoxicación en las minas de mercurio fue ampliamente conocido debido a las claras manifestaciones clínicas. Así, Bernardino Ramazzini (1633-1714), en su famosa obra *De morbis artificum*, señalaba que “es de las minas de mercurio de donde sale la maldición más cruel de todas las que tratan y destruyen a los mineros” (Ramazzini, 1964, p.21). Allí Ramazzini citaba a Gabriele Falloppio (1523-1568), quien, en su tratado *Metales y otros minerales*, planteaba que los mineros de mercurio pueden resistir apenas tres años, y a Michaelis Ettmüller (1644-1683), quien en su libro *Mineralogia* informaba que dentro de cuatro meses los intoxicados pueden presentar parálisis de las extremidades o generalizada, y vértigo, causados por los “espíritus mercuriales” (Ramazzini, 1964, p.21). En tiempos remotos el nombre dado a esta intoxicación fue hidrargirismo, debido a la palabra griega antigua *hydrargyros* (ὑδράργυρος) para designar el mercurio; será la que se usará en este escrito dado que así era conocida en la mina estudiada.

Esta enfermedad se caracteriza por eretismo, temblor y estomatitis, y la historia muestra que es frecuente en las minas de mercurio de todo el mundo. Es un tema bien conocido en minas antiguas como son las de Almadén (España), Huancavelica (Perú), Idrija (Eslovenia), entre otras (Nriagu, 1994), donde el mercurio se presenta en forma de cinabrio (HgS, combinación de mercurio y azufre). En el caso de las escasas minas de mercurio nativo (llamado así cuando se encuentra en forma líquida y sin mezclas con otros elementos), la exposición al metal pesado es mucho más alta debido a la mayor facilidad de evaporarse dentro de los socavones y generar ambientes de muy alta exposición (Kobal, Dizdareviĉ, 1997). En todos estos contextos el eretismo suele ser lo primero en aparecer y se caracteriza por cambios severos del comportamiento o personalidad; el temblor más frecuente ocurre en los miembros superiores cuando se hace un movimiento hacia un objetivo (temblor intencional), mientras la estomatitis se manifiesta por salivación excesiva y sensibilidad de las encías (Buckell et al., 1946).

En este estudio se argumenta que la labor de explotación en la mina de mercurio nativo de Aranzazu (Caldas, Colombia) estuvo atravesada por una alta ocurrencia de hidrargirismo que se presentó en unos pocos años. Este evento ocurrió poco después de la expedición del Código Sustantivo del Trabajo, en 1950 (decreto n.2.663), que señalaba que los administradores de las minas debían regular la contratación de los mineros y ofrecerles óptimas condiciones de seguridad laboral (Colombia, 1950). Antes del cierre de la mina, la enfermedad se trató de manera empírica con medicamentos quelantes (antagonistas de metales y metaloides que favorecen la excreción del cuerpo), de acuerdo con las pautas posológicas definidas por el médico oficial de la compañía. Si bien la prevención, diagnóstico y tratamiento del hidrargirismo se basó en literatura científica obtenida de diversas minas de mercurio alrededor del mundo, en gran medida fue una terapéutica experimental dado que en esa época no estaba estandarizada la forma de uso de los quelantes.

## Estudios historiográficos de la enfermedad ocupacional

A principios del siglo XXI, Diego Armus (2002) planteó que la enfermedad ha comenzado a ganar un terreno destacado en la historiografía latinoamericana por la fragmentación de las grandes corrientes historiográficas y por la preocupación que las ciencias sociales y humanas han tenido con la enfermedad como un objeto de investigación en la historia de la medicina y la de la salud pública. Este autor plantea que la historia de la enfermedad se ha desarrollado a partir de tres tópicos: dimensión social y política de las epidemias; las influencias externas en el desarrollo médico-científico y en las políticas de salud pública de la región; y los usos culturales de la enfermedad. Un grupo de estudios se ha enfocado en las enfermedades de los trabajadores, donde se enmarca este estudio.

Grandes aportes a la historia social de la enfermedad ocupacional, de los trabajadores y de la salud ocupacional se deben a pioneros como George Rosen (1985) y Paul Weindling (1985), que han fundamentado varios de los estudios hechos en diferentes países. En España, por ejemplo, Cohen y Ferrer (1992) analizaron enfermedades como anquilostomiasis y silicosis en zonas de influencia minera, y Menéndez Navarro (1992) se dedicó a explorar la relación entre trabajo, salud, enfermedad y asistencia a los mineros intoxicados en la mina de mercurio de Almadén. Sin duda los estudios en esta mina son fundamentales para comprender lo ocurrido en Aranzazu, aunque considerando las diferencias marcadas por las distintas formas químicas de presentación del mercurio. Esto contrasta con los estudios en América Latina donde han recibido mayor atención las temáticas institucionales y sobre discursos hegemónicos de la actividad médica tanto en Brasil (Lacaz, 2007; Almeida, 2008) como en Argentina (Grimberg, 2000).

En Colombia se ha escrito sobre la historia de la enfermedad entre algunos grupos de trabajadores. Sin embargo, como generalidad, los temas de salud no eran prioritarios en las luchas de los movimientos de trabajadores; se tendía a protestar por la escasa atención médica, y los mecanismos de compensación cuando ocurrían lesiones o enfermedades laborales (Torres, 2022, p.67). Algunos de estos estudios se enfocaron en los trabajadores de la industria petrolera (Quevedo, 1996; Quevedo et al., 2004; Luna-García, 2010), la agricultura cafetera (García, Quevedo, 1998; García, 2007; Urrego Mendoza, 2016), del ferrocarril (Restrepo, 2004) y de empresas mineras. Sobre este último escenario laboral, de interés en este trabajo, se ha estudiado la uncinariasis y la tuberculosis entre trabajadores de minas de oro en Antioquia (Gallo Vélez, 2010; Gallo Vélez, Márquez Valderrama, 2011a, 2011b; Ossa Viana, 2019). Como se puede ver, gran parte de los estudios sobre historia de la enfermedad entre trabajadores en Colombia se enfocan en enfermedades infecciosas, la mayoría catalogadas como “enfermedades tropicales”, presentes en los entornos donde se realizaban los trabajos. Esto es consistente con la literatura especializada en historia de la salud pública latinoamericana (Cueto, Palmer, 2015).

Por su parecido con el tema de este trabajo, los estudios sobre historia de la enfermedad respiratoria entre mineros resultan de especial interés, pues conlleva directamente a la enfermedad ocupacional, asociada a un agente presente en el ambiente laboral, y objeto de estudio de la medicina del trabajo (Kennedy, 1994). Esta línea de investigación ha sido desarrollada principalmente por Oscar Gallo Vélez (2010) en los estudios sobre trabajadores de empresas mineras, donde se describe la silicosis haciendo especial énfasis en las dificultades de diagnóstico y relacionamiento con el trabajo. Los estudios se enfocan en la mina de oro “El Zancudo”, localizada en Titiribí, Antioquia, donde las enfermedades respiratorias tenían una alta ocurrencia. La importancia histórica de esta mina en gran medida se relaciona con su relevancia económica durante más de un siglo. Utilizando registros hospitalarios y de las defunciones en Titiribí, entre 1865 y 1948, se concluye que las enfermedades respiratorias eran muy frecuentes, y que los médicos tendieron a asociarla más con tuberculosis; solo a comienzos de la segunda mitad del siglo XX empezaron a reconocer la silicosis entre los mineros (Gallo Vélez, Márquez Valderrama, 2011a, 2011b). Este sesgo en el diagnóstico médico debe entenderse por el momento epidemiológico de la primera mitad del siglo XX en el país, que incrementaba rápidamente la ocurrencia de la tuberculosis (Idrovo, 2001), así como las disputas similares que ocurrieron en otros lugares del mundo donde la presencia de tuberculosis pulmonar permitía ocultar la silicosis, de manera intencional o no intencional, en gran medida facilitada por la evolución crónica de la enfermedad (McIvor, Johnston, 2016).

Es precisamente en esta línea de estudios sobre la historia de la enfermedad ocupacional y de la medicina del trabajo que se enmarca el estudio de la intoxicación en la mina de mercurio nativo en Aranzazu. La reconstrucción de esta historia permite evidenciar la alta ocurrencia de hidrargirismo de aparición aguda, lo cual no ha sido observado en ningún otro hecho similar en el país. De hecho, pese a que en Colombia existe una alta exposición de mercurio en el aire en actividades de extracción de oro, catalogada como la mayor exposición per cápita a nivel mundial (Cordy et al., 2011), la ocurrencia de hidrargirismo es muy baja (Zapata Díaz, Mesa Arango, Berrouet Mejía, 2020). Estudios recientes sugieren que el antagonismo que genera el selenio de origen geológico (Varona-Uribe et al., 2023), muy alto en algunas regiones del país (Ancizar-Sordo, 1947) y también presente en varios alimentos (Córdoba-Tovar et al., 2022; Díaz et al., 2023) genera una protección contra la intoxicación con metales o metaloides. Tampoco se encuentran muchos casos asociados con la exposición a mercurio orgánico, por ejemplo, por ingesta de pescado (De Miguel et al., 2014), pese a que sí existen múltiples síntomas y signos inespecíficos entre la población expuesta.

Esta investigación se realizó bajo el entrecruzamiento de diversas fuentes primarias ubicadas en archivos y bibliotecas colombianas. En el Archivo General de la Nación se logró acceder a las licencias mineras autorizadas por el Ministerio de Minas y Petróleo (1953-1970) – actualmente Ministerio de Minas y Energía – y a la correspondencia del presidente de la República de Colombia con sus ministros de Minas. En la Biblioteca Luis Ángel Arango y la Biblioteca Nacional se consultó *La Patria*, el periódico de mayor tiraje en el departamento de Caldas, donde se encontraba ubicada la mina de mercurio, y los boletines informativos del Ministerio de Minas. En la biblioteca del Servicio Geológico Nacional se encontraron planos e informes técnicos sobre la mina “La Esperanza” y, en el municipio de Aranzazu, se tuvo acceso a documentos administrativos de la alcaldía y concejo municipal. Sin embargo, queremos subrayar que la mayor riqueza informativa se encuentra en las 15 entrevistas que se realizaron a ex trabajadores de la mina o sus respectivos familiares; información que posteriormente fue contrastada con los soportes documentales encontrados en los mencionados archivos. También se tuvo acceso a material audiovisual del colectivo cultural independiente “Aranzazu al día” y a las entrevistas que ellos realizaron en los últimos diez años. Este tipo de información fue organizada y sistematizada en el *software* libre Zotero, el cual permitió recopilar, citar y sincronizar cientos de datos junto con la discusión historiográfica. Finalmente, el estudio siguió los lineamientos para la investigación con humanos, definidos en la normatividad colombiana y en la Declaración de Helsinki, y fue avalado por el comité de ética en investigación científica de la Universidad Industrial de Santander el 7 de junio de 2019.

## Aranzazu y la mina “La Esperanza”

En Colombia existen diversas regiones con yacimientos de cinabrio, que incluyen mercurio en bajas proporciones, como Aguadas, El Limón (La Merced) y Samaria (Salamina), todas en el departamento de Caldas (Brooks, 2012), los cuales eran usados para la extracción de oro desde tiempos precolombinos (Brooks, Schwörbel, Castillo, 2011; Brooks, Bermúdez Restrepo, Cadena, 2016). De acuerdo con resultados de medición de mercurio en cabello en muestras prehispánicas (Idrovo et al., 2002) y de análisis históricos sobre la metalurgia desarrollada en esos momentos (Idrovo, 2005), al parecer, la exposición a mercurio era mínima. Con “el proceso o método de patio”, puesto en práctica en los territorios dominados por España durante el siglo XVI, se inició la amalgamación de mercurio con oro y plata (Nriagu, 1993, 1994). De esta manera, durante la época colonial, el mercurio usado en Nueva Granada (hoy Colombia) provenía de las minas de Almadén, España (Higueras et al., 2011), o de Huancavelica, en Perú (Brown, 2001; Cooke et al., 2009). Todas las actividades en Colombia que requerían usar mercurio dependieron, desde esa época, de la importación procedente de otros países, y solo cambió temporalmente mientras la mina de Aranzazu estuvo activa.

El municipio de Aranzazu fue fundado en 1853, limítrofe con los municipios de Salamina, Filadelfia, Marulanda y Neira, y ubicado a unos 50,5km de la capital departamental, Manizales (Figura 1). Durante las décadas de 1910 y 1930, tuvo un lugar privilegiado durante el auge del cultivo de café especialmente desde que se construyó un teleférico, o cable aéreo, usado para transportar carga y personas entre Aranzazu y Manizales, que funcionó entre 1928 y 1942. En 1922 se instaló el alumbrado público (Valencia Llano, 1996). Ello denota la importancia que tenía la ubicación del municipio para el transporte del café, la zona de mayor producción nacional de aquella época.

**Figura 1:** : Mapa con la ubicación de la mina de mercurio “La Esperanza” (Lozano, Pérez, Vesga, 1984)

La mina de mercurio “La Esperanza” se ubicaba en una zona boscosa de la cordillera central de los Andes, en la vereda El Manzanillo, jurisdicción de Aranzazu en el departamento de Caldas, Colombia. El territorio incluye montañas, valles y mesetas, con altitudes que varían entre 1.400 a 3.500msnm. Desde el punto de vista geológico, es una zona de transición entre la corteza continental y la corteza oceánica con intensos plegamientos, fallamientos, levantamientos e intrusiones batolíticas de gran magnitud, lo que conlleva a una gran complejidad estructural y estratigráfica (González Iregui, 2001). La anomalía positiva para el mercurio está relacionada con el sistema de fallas Romeral, estando la mina “La Esperanza” ubicada en la región más austral de este cinturón geológico (Lozano, Pérez, Vesga, 1984). La extensión de la mina rondaba las mil hectáreas, bañadas por las quebradas La Honda, Dantas y El Sargento, en un terreno de constantes deslizamientos de tierra, lo cual dificultaba el proceso de extracción del mineral (Licencia 1540, 17 mar. 1959).

En su historia se pueden establecer dos etapas identificadas por la forma de extracción del metal, lo cual tenía directas consecuencias en el nivel de exposición a mercurio en el aire y, por tanto, en los efectos adversos en la salud de los trabajadores. La primera etapa, entre 1948 hasta 1961, se caracterizó por tener explotación a cielo abierto con herramientas básicas. En esta etapa se pueden contar los siguientes momentos, de acuerdo con la compañía minera encargada de las labores de extracción: Roberto Botero, Consorcio Minero y Walter Ringueski (1948-1956), Borrero y Robledo (1957) y Chocó Pacífico (1958-1960). La segunda etapa, desde 1962, se caracterizó por el trabajo subterráneo y la participación del Consorcio Mineros Colombianos, un gremio de abogados especializados en derecho minero que obtenían las licencias por parte del Ministerio de Minas y Energías, y luego subarrendaban la administración de la mina. Con este método diversas compañías mineras lideraron la extracción de mercurio: Merco – Mercurio Colombiano – (1963-1965), Compañía Nueva Esperanza y Southern Union (1965-1970), Américo Marán (1970-1971) y Compañía Mercurífera de Caldas (1972-1975) (Lozano, Pérez, Vesga, 1984).

En 1948, en la propiedad del señor Roberto Botero, campesinos descubrieron mercurio nativo en medio de piedras de cuarzo, calcita y pirita con leves impregnaciones de cinabrio (Lozano, Pérez, Vesga, 1984). Es importante señalar que esta forma de presentación del mercurio es diferente al observado en las minas de cinabrio, como son la de Almadén, Huancavelica y la mayor parte de Idrija. Esta característica, poco frecuente en la geología mundial, implicaba una extracción menos tecnificada, mucho más rápida y con una menor inversión económica. La singularidad de la mina de Aranzazu solo puede compararse con las minas de mercurio nativo de Quicksilver Creek en Victoria Central, Australia (McQueen, 2011), y las de Clear Creek en el condado San Benito, California, en los Estados Unidos de América (Dunning et al., 2005). Estas dos minas han sido cerradas debido a su alto potencial tóxico y consideradas amenazas geológicas (Abbas et al., 2015).

Sin duda, entre 1948 y 1960, los mineros eran los mismos campesinos de Aranzazu y de los pueblos vecinos, quienes trabajaban en turnos diurnos con pica y pala y, a cielo abierto, buscaban el mineral abriendo grandes hoyos, debajo de las piedras y de los mismos árboles. Este trabajo en superficie no repercutía directamente en intoxicaciones a pesar de que la informalidad del trabajo conllevaba a que no usaran cascos, guantes ni botas apropiadas, incumpliendo con ello las medidas de salud ocupacional que se planteaban en el Código Sustantivo del Trabajo. De todos modos, fue en este periodo que se construyó el campamento con sus respectivos comedores y los catres para el descanso de los trabajadores, hechos a base de guadua y zinc (Orozco, mar. 2023).

A finales de la década de 1950, el trabajo en la superficie se fue transformando debido a la construcción de los primeros cuatro socavones (Figura 2); sin embargo, un informe técnico ingenieril de 1959 señalaba que

**Figura 2:** : Croquis del yacimiento de cinabrio de “La Esperanza” (Hubach, 1951)

no se han levantado planos de las excavaciones con sus localizaciones, cortes, direcciones y pendientes, lo cual hace incierto el resultado positivo de tales trabajos. El sostenimiento de estos túneles es difícil, por la calidad de sus respaldos y la porosidad de su suelo; la escasez casi absoluta de madera agrava aún más este problema (Informe…, 1959, p.4).

El primer túnel construido era el de mayor expectativa para los administradores de la mina por sus magníficas posibilidades de rendimiento y por la alta cantidad de mercurio libre y esquistos mineralizados de cinabrio que se hallaban, aunque la porosidad del terreno implicaba constantes derrumbes. Obedeciendo a situaciones coyunturales, fueron construidos los socavones dos, tres y cuatro con “la intención infructuosa de reencontrar el filón del túnel n.1” aunque los resultados no eran del todo satisfactorios por las dificultades del terreno (Informe…, 1959, p.3).

Ya en la segunda etapa, entre 1964-1967, se construyeron los túneles cinco y seis, los cuales tenían en promedio de “cien, 150 metros” (Naranjo, mar. 2023). Al final, todos los socavones contaron con alumbrado, rieles para el desplazamiento de las rocas y ventilación eléctrica dirigida por tubos de plástico (Lozano, Pérez, Vesga, 1984). En esta etapa el trabajo subterráneo se volvió más especializado y el pago que recibían los mineros dependía de las actividades realizadas. Los trabajadores encargados de las actividades en la superficie obtenían sesenta pesos semanales, mientras los responsables de ingresar en los socavones percibían entre cuatrocientos a quinientos pesos semanales. Sin duda, era una cifra muy superior a la obtenida en las actividades agrícolas, que era de siete a ocho pesos por jornal (Orozco, mar. 2023). En síntesis, trabajar en la mina “La Esperanza” repercutía en un aumento salarial en la vida de estos mineros que crecía exponencialmente cuando se desempeñaban funciones debajo de la tierra.

El trabajo en los socavones se realizaba en grupos de cuatro mineros, en turnos entre seis a ocho horas; dos de ellos trabajaban picando el material rocoso con pequeñas picas, y los otros dos perforaban el túnel con dinamita; el peligro de esta actividad fue evidente en 1966, cuando hubo una explosión que tuvo como consecuencia que dos trabajadores resultaran heridos (Heridos…, 4 jul. 1966). El material rocoso era retirado del túnel por los cocheros y transportado a la tolda de lavado, la máquina más grande de la mina, con la cual se limpiaba la roca con agua a presión, permitiendo separar la roca de los materiales inutilizables como la arena y el barro (Figura 3). Finalmente, la roca era llevada a una máquina trituradora de mandíbula, encargada de convertirla en polvo y el mercurio era conducido a una pequeña tolva con canales para envasarlo en botellas de acero (Figura 4) (Orozco, mar. 2023; Gallego, mar. 2023).

**Figura 3:** : Mineros de la compañía “La Esperanza” trabajando en superficie (Duque, s.f.a.)

**Figura 4:** : Trabajadores de la compañía “La Esperanza” batiendo el mercurio (Duque, s.f.b.)

La cantidad del mercurio extraído en la segunda mitad de los años 1960 era alta. Como ejemplo, se sabe que, en 1969, la mina produjo 23.734 libras de mercurio, que fueron distribuidas a Bogotá y Medellín (López Agudelo, 1971, p.18). Sin embargo, las condiciones de trabajo no eran adecuadas, pues “era un pantanero y olía feo, era puro barro” (Soto, mayo 2023). En principio, los trabajadores de la mina en Aranzazu no tenían las condiciones básicas de salud ocupacional que les permitiera reducir el nivel de intoxicación, en la misma medida que “el campamento está localizado en un punto inadecuado puesto que queda más bajo que los actuales trabajos y por lo tanto los obreros están sometidos a una intensa contaminación fuera de las horas de trabajo ya que en su jornada están sometidas a una contaminación aún mayor” (Morer, Nicholls, 1960, p.22).

Si bien no hay datos de la exposición a mercurio que ocurría dentro de los socavones, la evidencia de minas similares puede brindar información útil. Por ejemplo, en la mina de Idrija, Eslovenia, cerca del 70% del mercurio se encontraba en forma de cinabrio. El restante 30% como mercurio nativo. Precisamente, en estos lugares con mercurio nativo, cuando no había ventilación, las concentraciones de mercurio eran extremadamente altas, llegando incluso a superar los 3mg/m^3^ en el aire, lo cual se asociaba con un alto número de casos de hidrargirismo (Kobal, Dizdareviĉ, 1997). Algo similar ha sido descrito en la mina de Almadén, España, donde casi la totalidad del mercurio se encuentra como cinabrio; allí las concentraciones de mercurio en el aire podían llegar hasta 2,26mg/m^3^ en los lugares donde se perforaba la roca (García Gómez et al., 2007). Las descripciones del poco tiempo requerido para que se observaran los casos de hidrargirismo evidencian la muy alta exposición en Aranzazu. Nótese que Alfredo Menéndez Navarro (1996, p.151) describe que, en la mina de Almadén, los temblores y la estomatitis mercurial se presentaban “como un proceso de tipo crónico”, lo cual contrasta con las pocas semanas que laboraban los mineros en Aranzazu antes de presentar los signos de intoxicación mercurial.

Sin embargo, el mismo proceso de extracción del mercurio traía consigo una constante pérdida del mineral. Los geólogos visitantes de la mina, en la década de 1950 y 1960, resaltaban que la maquinaria usada era especializada para la extracción de oro y por ello “el gran defecto de esta pequeña instalación es que se tritura demasiado grueso para liberar todo el mercurio nativo contenido. Es difícil saber exactamente cuánto mercurio se pierde en los botaderos del molino, pero parece que se puede avaluar está perdida en un 20% a 30% del mercurio” (Morer, Nicholls, 1 dic. 1958, p.9). Otros autores señalaban que el porcentaje de pérdidas llegaba al 50%, en tanto “se está dilapidando riqueza aproximadamente igual a la que se beneficia” (Informe…, 1959, p.8). Precisamente, la pérdida de mercurio permitió que el trabajo del barequero apareciera en las quebradas El Chupadero y El Roblal, adyacentes a la mina. Los barequeros eran trabajadores mineros de subsistencia que aprovechaban los desechos mineros para recoger de manera rudimentaria el mercurio, lo cual, era conservado en botellas de vidrio (Alegría, mar. 2023). A pesar de las condiciones precarias en las que laboraban es poco probable que la exposición a mercurio fuese alta dado que el trabajo al aire libre les protegía de las grandes concentraciones del mineral. Asimismo, los barequeros incluían una mayor heterogeneidad de trabajadores, en tanto participaban menores de edad y mujeres, a diferencia de los trabajadores de la mina que eran solo hombres adultos (Martínez, mayo 2023). Aunque las ganancias eran mucho menores de la que recibían los mineros de la mina, los barequeros recolectaban el suficiente mercurio como para mejorar las condiciones de vida de sus familias.

## El hidrargirismo agudo: ocurrencia y presentación clínica

En Aranzazu, los síntomas del hidrargirismo ocurrían de acuerdo con los niveles de exposición al mercurio. La mayor exposición se presentaba en los trabajadores que laboraban dentro de los socavones, quienes en unos pocos días empezaban a presentar manifestaciones clínicas de hidrargirismo, seguidos de los trabajadores que tenían actividades en la superficie y, finalmente, los barequeros que tenían una exposición baja. Según los testimonios de los mineros, los primeros síntomas consistían inicialmente en la pérdida del apetito, dolores de cabeza y de los huesos, cansancio y parestesias, los cuales son compatibles con el eretismo mercurial. En muchos casos, estos síntomas eran tan comunes que las mismas señoras encargadas de la cocina identificaban a los enfermos a simple vista (Salazar, mayo 2020). Y ellas mismas avisaban a los administradores de la situación física del minero. Cuando la exposición aumentaba, los síntomas físicos y mentales del enfermo se agravaban. Por lo general, los mineros sufrían nauseas, diarrea, mal aliento, dolores de encía y los dientes recibían una coloración pardusca. Jesús María Álzate (mar. 2023) recordaba que se “le aflojaba la dentadura. Porque yo, imagínense, que (yo) empezó(cé) a dañarse mi dentadura y ya a los 16 años ya me tocó colocar una prótesis, una caja”.

En cuestión de días aparecía el signo evidente del hidrargirismo: el temblor intencional. Estos temblores se caracterizaban porque sobrevenían de forma ondulatoria, interrumpiéndose durante unos minutos, aunque se producían de manera tosca y fuerte para el individuo. El movimiento empezaba en los dedos de las manos, labios y párpados para transitar de manera progresiva a las extremidades (Gutiérrez, 1997). Estos temblores impedían que los trabajadores desarrollasen sus actividades diarias con plena libertad. Así, por ejemplo, Damaris – hija de un minero – recordaba que su padre requería ayuda para desarrollar las actividades básicas de la vida diaria como usar los cubiertos, tomar el desayuno y bañarse (Soto, mayo 2023). Reinaldo señalaba que había muchos enfermos en la mina:

Gente que les cogía esa tembladera en el cuerpo y no se hallaban ni parados. No se sostenían ni parados. Allá le agarraba a uno esa cosa y uno no era capaz de comer. Uno cogía la cuchara aquí y cuando llegaba a la boca yo no tenía nada. No había nada en la cuchara. Y pa’l tinto era igual. Yo me acuerdo que cuando me agarró eso yo me fui para Aranzazu y me iba a tomar un tinto. Y si no iba nadie conmigo no me lo podía tomar (Orozco, mayo 2023).

Otro de los síntomas asociados al hidrargirismo eran las presentaciones neuropsiquiátricas. Históricamente eran llamadas “eretismo mercurial” (Parsons, Percival, 2005), manifestadas por los rápidos cambios que experimentaba el individuo en su personalidad. Entre otras cosas, aparecían trastornos psíquicos como depresión, llanto inmotivado, pérdida de memoria, psicosis maniaco-depresivas e irritabilidad (Gutiérrez, 1997). La señora Marina Gómez (abr. 2023) relataba que su esposo era incapaz de conciliar el sueño con lo cual mantenía unos niveles altos de susceptibilidad emocional, llevada a los extremos entre la tristeza y la alegría, con fuertes lapsos de depresión. Sin embargo, debemos señalar que el eretismo por intoxicación mercurial quizá no fue muy llamativo en Aranzazu, dado que la población tiene una de las mayores ocurrencias de trastorno afectivo bipolar en Colombia asociada con la endogamia de sus habitantes desde finales del siglo XIX (Bedoya et al., 2006; Jordán-Quintero et al., 2022). Por tanto, quizá la manifestación más llamativa fueron los temblores que los entrevistados relacionan claramente con el trabajo en la mina de mercurio.

Ahora bien, esta combinación de síntomas generada por la intoxicación con mercurio llevó a grave deterioro de la salud de varias personas; por un lado, porque en los primeros años de funcionamiento de la mina se desconocía el tratamiento médico que se debía llevar a cabo para tratar el hidrargirismo y porque los mineros acostumbraban a consumir grandes cantidades de bebidas alcohólicas en medio de la debilidad corporal. Rubiel Álzate (mar. 2023) señalaba que “la gente se iba secando, se iba a hacer [convirtiendo] en huesitos y se veían en las cantinas del pueblo”. Asimismo, Renet Giraldo (mar. 2023), hijo del propietario de la fonda más cercana a la mina, recordaba que los mineros “venían acá [fonda] a tomar cerveza y había que servirle el vaso a la mitad porque si no se les regaba, les daba tembladera en la mano”.

Para calcular el número de mineros intoxicados por hidrargirismo seguimos los testimonios de extrabajadores y del médico oficial de la mina, Hernando Marín Maya (1941-2021), contratado en 1967 por la junta administrativa del Consorcio Minero para tratar la enfermedad. Marín señalaba que cuando él inició labores cerca de 95% de los mineros presentaban hidrargirismo, y que gracias a sus intervenciones bajaron en gran medida. En sus propias palabras, “el 95% tenían afectación de intoxicación con mercurio” (Maya, mayo 2020). Sin duda, la presencia de Marín Maya, desde 1967, marcó un antes y un después en términos de los casos de hidrargirismo. En los primeros años de trabajo en la mina no debió haber cambios significativos en la ocurrencia de casos de intoxicación, por lo que es posible que la ocurrencia fuese la que venía observándose durante el tiempo en que hubo actividades subterráneas. En contraste, en los momentos previos al fin de actividades mineras, en 1975, sí debió haber bajado significativamente la ocurrencia de casos de hidrargirismo. Otro ex trabajador, con labores administrativas en la mina, recuerda que, “si trabajaban cien hombres, yo quiero ver, había 25 enfermos” (García, mar. 2023).

Una aproximación al riesgo que tenían los trabajadores de ser diagnosticados con hidrargirismo requiere definir el total de trabajadores de la mina, lo cual puede ser estimado con lo afirmado directamente por varios trabajadores que laboraron en los últimos diez años de actividad extractiva. Uno de los ex mineros informaba que “ahí en ese tiempo que yo estuve allá habíamos 250 obreros” (Orozco, mar. 2023), lo cual fue confirmado por otros trabajadores que reportan que había “alrededor de cien trabajadores más o menos” (Naranjo, mar. 2023), y que los trabajadores eran “muchos, podrían tener más o menos cien o más porque trabajaban en turnos rotativos” (Soto, mayo 2023). Incluso el último de los inversionistas es consistente en el número de trabajadores, “yo llegué a tener hasta 120 allá” (Marán, jul. 2018).

Quizá uno de los motivos de las discrepancias en el número de trabajadores se relacionaba con la variedad de actividades realizadas, pues “eran muchos por ahí cien o más porque como eran tres turnos, había unos apalancando, otros cargando los cochecitos para bajar con ese material, otros moliendo, era mucha la gente porque como eso lo tenían que moler” (Agudelo, mar. 2023). Además, es evidente en los relatos de los ex trabajadores que el número de personas laborando en la mina no era constante; “sí, había muchos. Unos cincuenta, cien. Hay más o menos. Ya tenían maquinaria” (Álzate, mar. 2023). Esto sugiere que la llegada de la tecnología permitió una mayor producción y, quizá, una disminución del número de trabajadores en labores fuera de los socavones. En el periodo cercano al final de las actividades mineras, el número de trabajadores disminuyó: “Con don Américo había como treinta, ya era poquito. Pero allá hubo gente de ochenta y cien” (Agudelo, mar. 2023). Sin embargo, el número de trabajadores en las actividades subterráneas pudo ser más constante dada la imposibilidad de tener muchos trabajadores dentro de los socavones. “Éramos 25, estamos hablando más o menos de hace cuarenta años, yo creo que por ahí en el setenta. Por todos los socavones éramos 25” (Gallego, mar. 2023).

Los relatos anteriores permiten identificar el riesgo de ser diagnosticado con hidrargirismo. El total de trabajadores de la mina “La Esperanza” varió entre cien y 250, y los casos de hidrargirismo eran cercanos a una cuarta parte, lo que equivale a un número entre 25 y sesenta trabajadores aproximadamente. Además, no debe olvidarse la alta rotación de trabajadores, debido a la aparición del cuadro clínico de intoxicación observado apenas una o dos semanas después de iniciar labores, y al miedo de adquirir la enfermedad, especialmente entre los mineros trabajando en los socavones, sugiere que el riesgo de ser diagnosticado con hidrargirismo era cercano al 100% entre los trabajadores con labores subterráneas y cercano al 25% considerando todas las actividades de la mina. Estos casos deben multiplicarse por el número de años en que hubo extracción minera sin intervenciones de salud ocupacional, que puede ser mayor a una década. Con estas consideraciones y los testimonios de mineros y familiares se puede afirmar que el hidrargirismo en la mina de Aranzazu es uno de los eventos de intoxicación masiva de origen ocupacional con mayor ocurrencia en Colombia, por lo que se requieren más estudios para conocer el impacto en la salud de los trabajadores.

## Presencia médica y asistencia a los enfermos

A pesar de que la normatividad colombiana empezó a regular el trabajo minero desde 1915, cuando se expidió la ley 57, que obligaba la reparación por accidentes de trabajo y que se amplió a la prevención de la enfermedad con el Código Sustantivo del Trabajo, en 1950, la atención de los trabajadores de la mina de mercurio solo tuvo claras intervenciones de salud ocupacional en los últimos años de operación. Hasta 1966 no había un servicio médico contratado, y la atención a los mineros enfermos se hacía de manera informal en la botica de Aranzazu, motivo por el cual no se hallaron datos precisos sobre este tipo de atención. Sin embargo, la situación cambió en 1967, con la llegada del médico Marín Maya, quien fue contratado por la compañía Southern Union para organizar el servicio médico y orientar las medidas de salud ocupacional en la mina. Entre otras cosas, Marín Maya recopiló una extensa literatura sobre informes médicos y técnicos de las antiguas minas de Almadén y Huancavelica, que lo llevó a definir que la forma de combatir el hidrargirismo no era curándolo, sino previniéndolo. Esta era una tesis que en los años 1950 se discutía con fuerza en los círculos científicos españoles después del fracaso médico que implicaba la atención de los mineros enfermos (Manzanares, Montes, 2011).

Influenciado por los avances médicos en la mina de Almadén, Marín Maya planteó una estrategia de salud ocupacional para reducir los niveles de intoxicación de los mineros de Aranzazu. Inicialmente, se establecieron unas normas higiénicas de obligado cumplimiento por parte del personal minero; entre otras cosas, se prohibía fumar, consumir alcohol o llegar “enguayabado al trabajo” (veisalgia), y se insistía en mantener limpios los uniformes e implementos de seguridad. Las medidas fueron colgadas en carteles de madera y afiches en los espacios visibles del campamento, en tanto los individuos que no las cumplieran eran retirados de la mina (Maya, mayo 2020). Otra de las medidas instauradas era los exámenes médicos que se realizaban en el campamento tres días a la semana, los lunes, miércoles y viernes (Maya, mayo 2020). En principio, los mineros eran examinados de manera general con el objeto de identificar alguna anomalía que indicara la ocurrencia de hidrargirismo. Toda la información recopilada era trasladada a una historia clínica y los enfermos diagnosticados se les acompañaba en su proceso de recuperación. Básicamente, se recomendaba aumentar las dietas en proteína (con un alto consumo de carne de res y leche), se limpiaban las zonas lastimadas del cuerpo y, según la gravedad de la enfermedad, se prohibía el ingreso en los socavones y eran incapacitados por el tiempo que fuera necesario (Orozco, mar. 2023).

Sin embargo, estas atenciones médicas que recibían los mineros enfermos no surtían el mismo efecto para todo el personal. Damaris Soto relataba que su padre fue retirado de la mina una vez se enfermó y sus familiares se responsabilizaron de sus cuidados:

Cuando eso no existían el copito, entonces uno cogía un palillito y le ponía algodón y se tenía que limpiar diente por diente porque la ‘mercuriada’, eran como llenos de lama, los dientes eran flojitos, era como podrida la encía y se percibía el mal aliento, entonces uno debía coger diente por diente para limpiarlos y aplicarle el remedio (Soto, mayo 2023).

En ocasiones, los mineros enfermos no recibían ningún tipo de auxilio económico mientras estaban incapacitados y, por tanto, buscaban cambiar su suerte encontrando otro trabajo o inclusive migraban a otras ciudades (Gómez, abr. 2023). Este tipo de situaciones, como se señaló anteriormente, generaba una constante rotación en el personal minero y por ello es posible suponer una ocurrencia elevada de hidrargirismo que resulta difícil de documentar.

Para el tratamiento de los mineros que enfermaban de hidrargirismo se adecuó una enfermería con seis camillas, atendida por una enfermera dentro del campamento de la mina; en ella se cuidaba a los enfermos en su fase más grave o los que no tenían familia en la región. La atención duraba hasta que los individuos manifestaban mejoría para trasladarse a sus respectivas viviendas en las casas familiares o en los mismos catres de la mina (Orozco, mar. 2023). Por lo general, a los mineros enfermos se les aplicaba dimercaprol – Anti-lewisita británica (BAL) – y penicilamina, quelantes que apenas unos años antes se habían introducido en la terapéutica toxicológica. El BAL fue desarrollado por el Ejército británico durante la Segunda Guerra Mundial para combatir la lewisita, un arma química Nazi a base de arsénico (Waters, Stock, 1945). Este medicamento tenía como objetivo facilitar la expulsión del mercurio a través de la orina, aunque sus efectos adversos eran múltiples: hipertensión, taquicardia, nausea, vómito, sensación de quemadura en la boca y faringe, parestesia de manos, sensación de opresión o dolor torácico, lagrimeo, salivación excesiva, rinorrea, sudoración y dolor abdominal (Gerhardsson, Kazantzis, 2015). Por cierto, los primeros estudios con BAL en humanos fueron realizados en individuos con sífilis, principalmente de origen afro (Modell, Gold, Cattell, 1946). Los mineros de Aranzazu y sus familiares sabían de lo novedoso del tratamiento: “Mi papá ya pudo seguir el tratamiento, que llegaron una vez a la mina y entonces viendo esa situación experimental, mi papá fue de los primeros, por no decir el primero que se sometió a un tratamiento que le hicieron … y gracias a dios terminó sus días muy bien” (Soto, mayo 2023).

Por su parte la penicilamina, un quelante que resulta de la degradación hidrolítica de la penicilina, fue propuesta por Walshe (1956) como tratamiento de la intoxicación con mercurio teniendo como ventaja el menor número de efectos adversos en comparación con el BAL (Parameshvara, 1967). De hecho, solo en algunos individuos podía ocasionar reacciones de sensibilidad aguda como fiebre, rash cutáneo, discrasias sanguíneas y, en casos graves, daño renal (Gerhardsson, Kazantzis, 2015). Aunque no se tiene claridad la vía de administración y dosificación de los quelantes que se usaban en la mina, artículos de la época sugerían que el BAL era útil vía parenteral y la penicilamina podía aplicarse intramuscular (Pagnotto, Brugsch, Elkins, 1960). Reinaldo, un ex minero, relataba que le aplicaban en la enfermería “cuatro inyecciones diarias, dos por la mañana, dos por la tarde. Le ponían una en la vena y la otra muscular” (Orozco, mar. 2023), lo que quizá sugería el uso de ambos quelantes al mismo tiempo. Esto, siguiendo lo estipulado en artículos científicos debía hacerse tres veces en ciclos de nueve días, con intervalos de una semana entre uno y otro ciclo (Parameshvara, 1967). Al final, Reinaldo se recuperó del hidrargirismo al cabo de tres meses, más rápido del tiempo habitual.

En ocasiones, los mineros enfermos se agravaban tanto en los síntomas de la intoxicación que debían ser internados en centros hospitalarios en Manizales o Medellín. Si bien el hidrargirismo no suele llevar a la muerte, es probable que en algunos casos empeorara condiciones preexistentes que podrían agravarse. Por eso, se prohibió que los mineros enfermos ingresaran a los socavones y, en cambio, se estimulaban las actividades al aire libre y se insistía en la desinfección de las herramientas de trabajo como los uniformes, picas y palas. En todo caso se buscaba difundir prácticas de limpieza y cuidado en la vida cotidiana como no fumar y reducir el consumo de alcohol (Marán, mar. 2023).

## Consideraciones finales

En 1948 fue descubierto el mercurio nativo en Aranzazu (Caldas, Colombia) con lo cual se inició un trabajo de explotación del mineral al aire libre por parte de los campesinos de la región. Con la construcción de los primeros socavones y la llegada del consorcio mineros colombianos, en 1962, el trabajo en la mina se concentró en explotaciones bajo la superficie terrestre y en una extracción del mineral más tecnificada que en la década anterior. Dado que no hay evidencia del uso de elementos de protección respiratoria, la exposición al mercurio nativo debió ser muy alta dentro de los socavones, donde se concentraba el metal en el aire. Esto conllevó a que los trabajadores con actividades subterráneas presentaran hidrargirismo apenas unas semanas después de iniciar a laborar. Las actividades en superficie tenían menor exposición, pero el riesgo de enfermar se mantenía latente debido a que el proceso no incluía maquinaria apropiada; se adaptó la tecnología de la minería del oro que permitía importantes pérdidas del metal. Dado el número de trabajadores que se exponían a altas concentraciones de mercurio, la mina “La Esperanza” puede ser la empresa donde ocurrió uno de los mayores desastres de salud ocupacional en Colombia.

La llegada del joven médico Marín Maya, en 1966, mejoró radicalmente la situación. La identificación de la prevención del hidrargirismo como la principal intervención, mediante la definición de unas medidas de higiene y salud ocupacional que debían ser cumplidas por todo el personal, basadas en las experiencias de las minas de Almadén y Huancavelica, es sin duda un claro ejemplo de las actividades que promulgaba la salud ocupacional. Así, en la mina “La Esperanza”, se publicaron normas higiénicas prohibiendo fumar y beber alcohol e incentivando la desintoxicación de las máquinas y los uniformes. Además, se instaló un servicio médico de tres días a la semana, con el cual, a los enfermos se les recomendaba una dieta rica en carnes o se les aplicaba dosis de quelantes (BAL y penicilamina) para aumentar la excreción de mercurio del organismo a través de la orina. Estos medicamentos de uso experimental para la década de 1950-1960 fueron usados por primera vez en Colombia para tratar a los intoxicados de hidrargirismo de Aranzazu, teniendo resultados positivos. Ex mineros como Reinaldo Orozco se recuperaron en tres meses de la intoxicación, aunque no todos los enfermos lograron acceder al tratamiento. Muchos se habían retirado de la mina y dedicado a otras actividades económicas.
